# Oxytocin Effect on Collective Decision Making: A Randomized Placebo Controlled Study

**DOI:** 10.1371/journal.pone.0153352

**Published:** 2016-04-12

**Authors:** Uri Hertz, Maria Kelly, Robb B. Rutledge, Joel Winston, Nicholas Wright, Raymond J. Dolan, Bahador Bahrami

**Affiliations:** 1 Institute of Cognitive Neuroscience, University College London, 17 Queen Square, London, WC1N 3AR, United Kingdom; 2 Department of Experimental Psychology, University of Oxford, Oxford, OX1 3UD, United Kingdom; 3 Wellcome Trust Centre for Neuroimaging, Institute of Neurology, University College London, London, WC1N 3BG, United Kingdom; 4 Max Planck University College London Centre for Computational Psychiatry and Ageing Research, London, WC1B 5EH, United Kingdom; 5 Institute for Conflict, Cooperation and Security, School of Government and Society, University of Birmingham, Edgbaston, Birmingham, B15 2TT, United Kingdom; 6 Carnegie Endowment for International Peace, 1779 Massachusetts Avenue NW, Washington DC, United States of America; Beijing Normal University, CHINA

## Abstract

Collective decision making often benefits both the individuals and the group in a variety of contexts. However, for the group to be successful, individuals should be able to strike a balance between their level of competence and their influence on the collective decisions. The hormone oxytocin has been shown to promote trust, conformism and attention to social cues. We wondered if this hormone may increase participants’ (unwarranted) reliance on their partners’ opinion, resulting in a reduction in collective benefit by disturbing the balance between influence and competence. To test this hypothesis we employed a randomized double-blind placebo-controlled design in which male dyads self-administered intranasal oxytocin or placebo and then performed a visual search task together. Compared to placebo, collective benefit did not decrease under oxytocin. Using an exploratory time dependent analysis, we observed increase in collective benefit over time under oxytocin. Moreover, trial-by-trial analysis showed that under oxytocin the more competent member of each dyad was less likely to change his mind during disagreements, while the less competent member showed a greater willingness to change his mind and conform to the opinion of his more reliable partner. This role-dependent effect may be mediated by enhanced monitoring of own and other’s performance level under oxytocin. Such enhanced social learning could improve the balance between influence and competence and lead to efficient and beneficial collaboration.

## Introduction

Collaborative behaviour provides benefits to both the individual and the group [1]. Groups of cooperating individuals benefit when tackling social dilemmas [1–3] and promote common interests [4,5]. Groups also achieve collective benefit in perceptual tasks, outperforming the best individual member. However, cooperation is not always the chosen (or indeed the rational) strategy for interacting with others. Self-interest often trumps common interest, as group members make decisions aimed at maximizing their personal gain regardless of, and sometimes against, the group’s interests [4,6]. Collective decisions can also fail when participants fail to calibrate their contribution to the group with their own performance, with poor performers tending to be overconfident and competent performers tending to be underconfident [7–11].

The problem of calibrating one’s contribution to group performance is demonstrated by the equality bias heuristic, i.e. assuming that every group member is as competent or as reliable as everyone else [11]. Dyads engaged in a joint oddball detection task showed such bias, with better performers overweighting the opinions of their less competent partners during disagreements about the oddball location. This resulted in suboptimal dyadic oddball detection accuracy. A study using a similar experimental design to Mahmoodi et al. (2015) showed that administering exogenous testosterone swayed participants’ bias, rendering them more egocentric and less likely to change their mind during disagreements [12].Because testosterone’s egocentric discounting affected both better and worse dyad members, regardless of their performance level, it deteriorated group decisions and collective benefit. Can another intervention sway the weighting of other’s opinions to the other side, making them more allocentric, and will it have the same suboptimal effect dyadic performance as egocentric bias?

A likely candidate for shifting participants’ to the more allocentric side is the hormone oxytocin. Recent studies indicate that oxytocin can promote effective collaboration [13,14]. Exogenous oxytocin increases trust in an economic game [15], causing, on average, participants to send more money to a trustee, reinforcing the optimistic assumption that the trustee will return more than they invested, thereby increasing the benefit from social interaction. This tendency for greater optimistic risk taking in social contexts is also seen in an increased altruism under oxytocin [16,17]. De Dreu et al. [16] showed that, under oxytocin, participants tend contribute more to the public good and were willing to risk personal loss to that end suggesting a promotion of collaboration by reducing self-interest in social contexts. Finally, oxytocin was shown to enhance conformity to other group members [18,19], making participants more likely to align their views to the group’s. Social effects of oxytocin are not always positive, and under certain experimental designs oxytocin was shown to increase negative social feelings such as envy, gloating [20] and outgroup hostility [21]. The current experimental design does not include competition between participants, and we expected that the positive social effects of oxytocin, namely increased trust, altruism and conformity, would make the interacting partners more likely to take each other’s view into account, making them more allocentric.

Here we tested the hypothesis that oxytocin will make participants more allocentric, i.e. more likely to change their mind during disagreements. We further hypothesised that such allocentric bias will decrease dyadic performance, as the more competent member of a dyad will tend to follow the opinions of the inferior partner during disagreements. We employed a double-blind placebo-controlled design to examine a collective benefit accrued by pairs of participants engaged in a collective perceptual decision making task, similar to the one used to demonstrate equality bias [11] and egocentric discounting effect of testosterone [12]. In this task participants first searched for a visual oddball individually and then made a joint decision on trials on which they disagreed. It is then possible to examine individual and dyadic perceptual sensitivity, and extract additional measures of collaborative behaviour such as performance similarity [22,23] and a tendency for making egocentric joint decisions [12]. Importantly, and diverging from previous oxytocin studies, these measures are extracted from real-life dyadic interactions and disagreement resolution between two participants.

## Methods

### Participants

**Fig 1:**
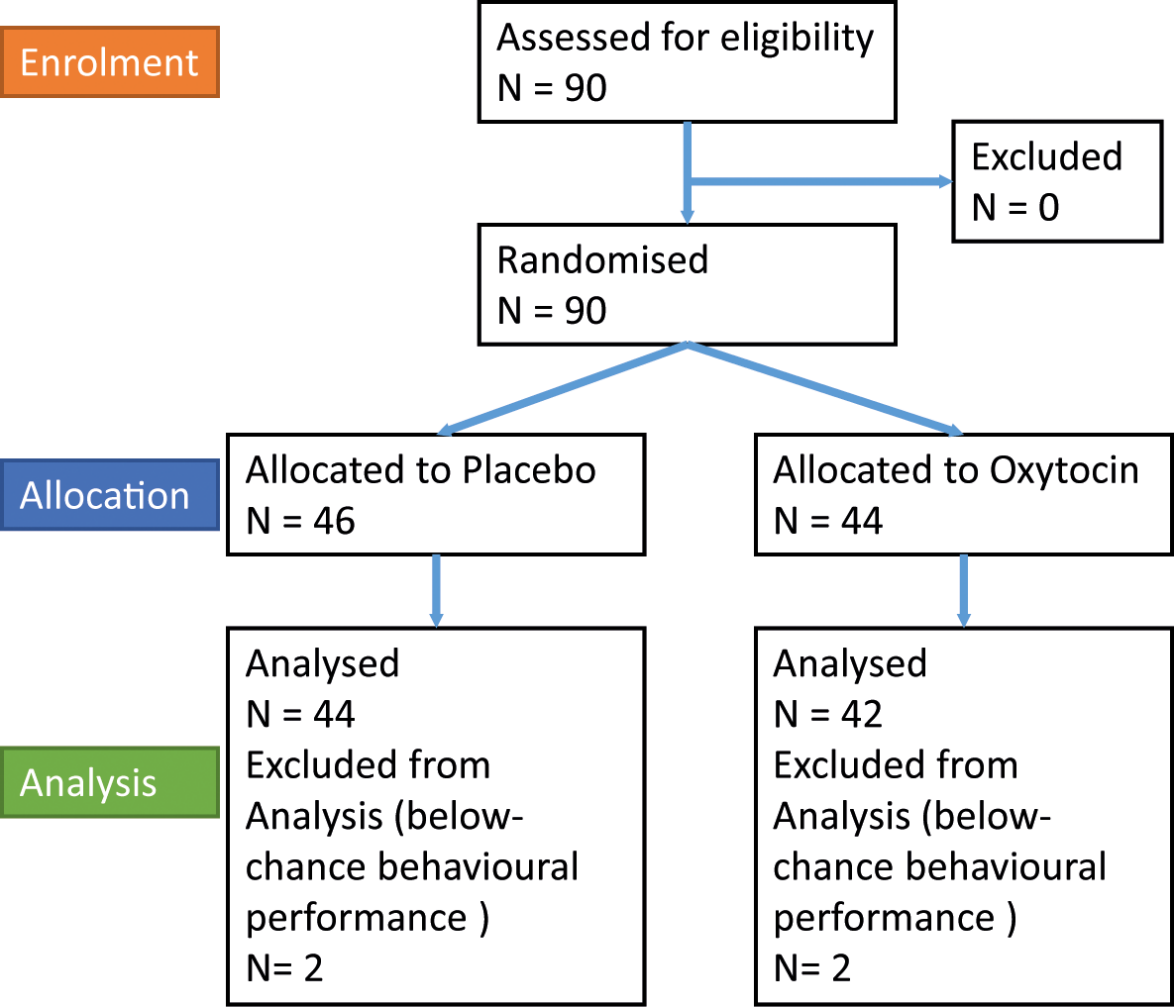
CONSORT flow diagram. Flow diagram of the progress through the phases of a parallel randomised trial of two groups [66].

We tested 90 healthy adult male participants (age range: 18 to 35; mean ± SD: 23.5±4.0), paired into 45 dyads. We estimated that a total of 43 dyads would be needed to detect a difference between groups, with a two-tailed α of 0.05 and a (1-β) of 0.80, in agreement with previous studies regarding the effect of oxytocin [15,16]. Two dyads were excluded due to below-chance behavioural performance of at least one of the dyad members (resulted in negative slope of the psychometric curve, see below)(Fig 1). These included 1 placebo dyads and 1 oxytocin dyads. Dyads were randomly assigned to either the oxytocin (oxytocin; 22 dyads, 44 male participants) or placebo condition (placebo; 21 dyads, 42 male participants), and treatment groups did not differ in terms of age (mean ± SD: oxytocin: 23 ± 3.8; placebo: 24 ± 4.5; t(84) = .69; p = 0.48) or body mass index (mean ± SD: oxytocin: 22.4 ± 2.4; placebo: 23.1 ± 3.1; t(84) = 1.24; p = 0.22). Given the potential for sex differences in oxytocin response [24–26] the sample was confined to men. Members of each dyad were unfamiliar to each other and the experimenter. Self-reports indicated that all 90 participants were healthy and had normal or corrected to normal visual acuity. Exclusion criteria for participants included neurological, endocrine, or psychiatric conditions, as well as nasal congestion or breathing difficulties. Participants were instructed to refrain from food, smoking, and drinking (other than water) for 2 hours before the experiment, and from alcohol and/or recreational drugs for 24 hours before and after. All gave written informed consent and were paid for attendance. The study was approved by the University College London Ethics Committee and the National Research Ethics Service Committee South East Coast—Surrey.

### Apparatus and stimuli

**Fig 2:**
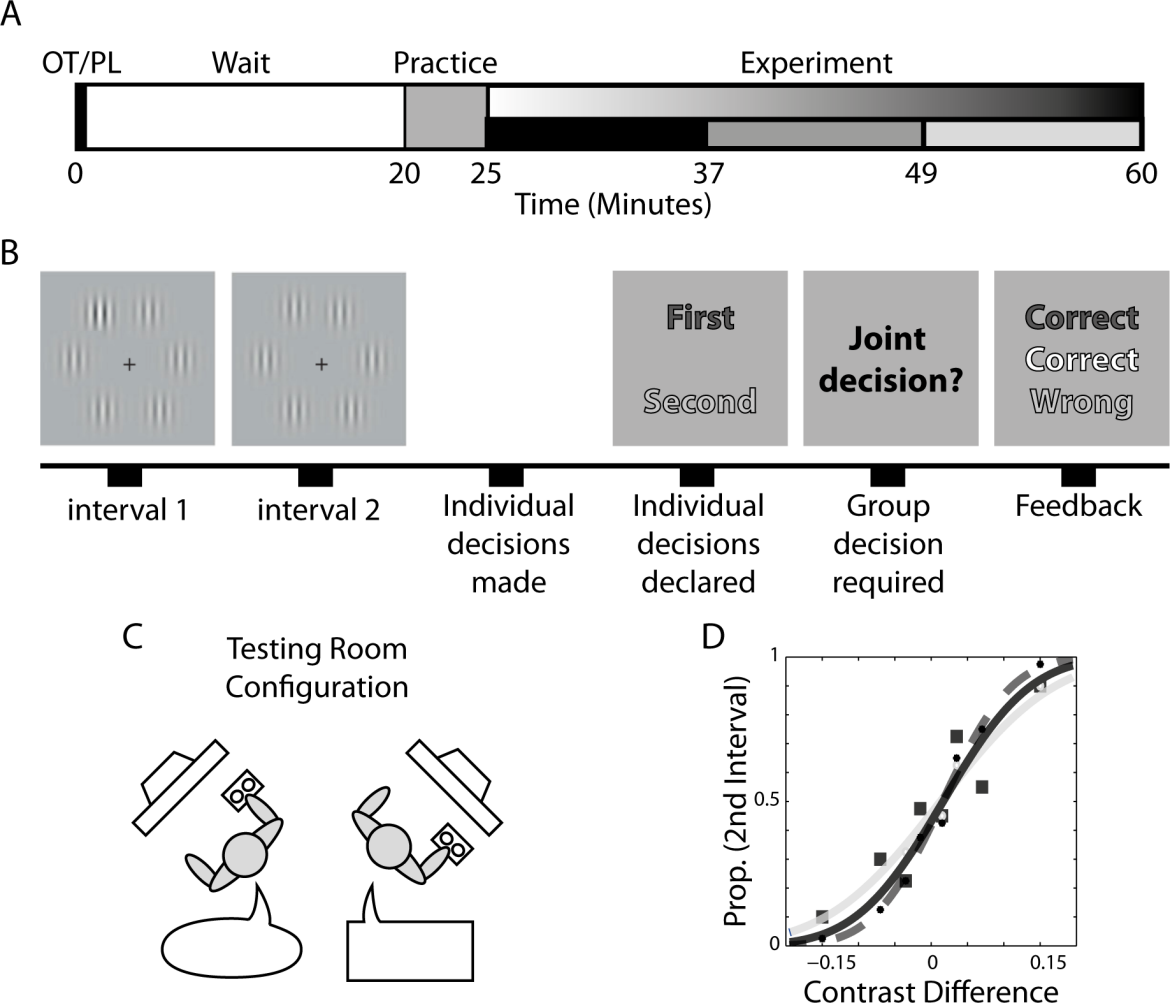
Experimental and task design. (A) Experimental timeline. The experiment started with intranasal self-administration of oxytocin or placebo (double blind). Participants waited quietly for 20 minutes and the performed a 5-minute practice block. The experiment proper started 25 minutes after administration. The experiment duration varied but lasted at least 35 minutes in all dyads, by which time the average of 224 were carried. (B) Pairs of male participants (dyads) performed together a 2-alternative forced choice oddball detection task. Visual stimuli consisted of six vertically oriented Gabor patches displayed equidistantly around an imaginary circle. One randomly selected interval contained the target of higher contrast. Participants indicated their individual decisions privately. If they disagreed, a joint decision was negotiated and announced. Feedback about accuracy was provided. (C) Participants sat in the same testing room, each viewing his own display. Display screens were placed on separate tables at a right angle to each other. (D) We measured the proportion of trials on which the observer reported that the target was presented in the second interval in different target contrast levels, and fitted a psychometric curve to these data. For each dyad three curves were fitted: two for the individual decisions made by each dyad member, and one for the dyadic decisions. Here we depict the psychometric curves fitted to one exemplar dyad. We present the better member data (dark square) and fitted curve (dark line), worse member data and fitted curve (light line and light circles), and dyadic data and fitted curve (dark dots and dashed line). In this case dyadic slope is steeper than the best member’s slope, indicating positive collective benefit.

Both dyad members viewed identical stimuli, presented on separate display monitors in the same room (Fig 2C). One dyad member responded using the keyboard, the other used the mouse. Both participants used their right hand.

The two display monitors (18”, 800×600 resolution, 4:3 aspect ratio, background luminance was 62.5 Cd/m^2^) were connected to the same graphics card, via a video amplifier splitter, and were controlled by the Cogent 2000 toolbox (www.vislab.ucl.ac.uk/cogent.php) for MATLAB (release 2013b, The MathWorks Inc., Natick, Massachusetts, United States).

The stimuli consisted of six vertically oriented Gabor patches (SD of the Gaussian envelope: 0.45 degrees; spatial frequency: 1.5 cycles/degree; contrast: 10%), displayed equidistantly around an imaginary circle (radius: 8 degrees). An ‘oddball’ target stimulus was generated by elevating the contrast of one of the six Gabor patches. The target contrast level (i.e., +1.5%, +3.5%, +7.0%, or +15%) varied randomly across trials, as did the interval and location in which the target occurred (Fig 2B).

### Randomization and Blinding

The assignment of placebo or oxytocin treatment was randomised using Matlab, and identical bottles for oxytocin and placebo were labelled with dyad number by BB. The experiments proper were run by UH and MK who were blind to the labelling. They recruited participants and assigned them to dyads based on availability, and provided the bottles to the dyad according to the dyad number, with no knowledge of the content of the bottles. Participants had no knowledge of the content of the bottle, but knew that they may contain oxytocin or placebo.

### Drug Administration Procedure

Participants self-administered either 40 IU of oxytocin (Syntocinon spray; Novartis) or placebo intranasally [27,28]. Appearance of the content of the bottles was identical for both group members. Participant spray instructions were as per those described by Guastella et al. 2013 [28]. Participants first ‘primed’ (pumped) the spray bottle until a fine mist appeared (2–3 pumps) then closed one nostril with one finger while administering spray to the other nostril. The nozzle was inserted approximately 1cm into the nostril (parallel to the nasal septum) at an angle of 45° (from tip of nozzle to horizontal plane). Upon delivery of one puff, participants inhaled and breathed in lightly. Delivery was then alternated between nostrils until 10 puffs had been administered. The spray was administered with head in the upright position, and at least 15 seconds were allowed between each re-administration to the same nostril.

Care was taken to make sure that the placebo spray matched the experimental oxytocin spray in all respects except the active ingredient. The placebo was prepared by the Royal Free Hospital Pharmacy Manufacturing Unit (Pond Street, London UK) to match the carrier of Syntocinon (the synthetic analogue of oxytocin). Both sprays were prepared according to the European Union guidelines on good manufacturing practice. The placebo and Syntocinon spray bottles were identical in appearance with the exception of a dyad number (the content associated with dyad number was unknown to the experimenter and participants). The correspondence between group number and chemical content of the bottles was recorded in look-up table prepared and maintained by a third party who was not involved in data collection.

After spray administration was complete, participants sat quietly for 20 minutes and spent the time reading a book or magazine. They had previously been advised about this waiting period. They were not allowed to interact with each other during the waiting time. 20 minutes waiting period was chosen in order to make sure that participants will have accomplished sufficient number of collective decisions (128) within 1 hour from oxytocin/placebo administration, when peak effect of oxytocin was expected (see below).

### Task Procedure

After the 20 minutes waiting period, participants performed the practice task (duration ≈ 5 minutes) followed by the experimental task (duration ≈ 36 minutes or more). The experimental timeline is illustrated in Fig 2A. Both dyad members performed one practice block (16 trials) and twenty experimental blocks (320 trials, lasting at least 36 minutes) of a 2-alternative forced choice visual perceptual task [22]. We assumed that dyads will be able to finish at least 128 trials within 35 minutes (i.e. within 1 hour from oxytocin/placebo administration), sufficient number of trials for psychophysical function fit (see below). Each trial started with a black central fixation cross (0.75 degrees visual angle). Following a variable time period, drawn uniformly from the range 500–1000 ms, two successive observation intervals were displayed. The stimulus duration in each interval was 85 ms, and the intervals were separated by a blank display lasting 1000 ms. An ‘oddball’ target (Gabor patch with higher contrast grating) occurred either in the first or second interval.

After the second interval, the fixation cross turned into a question mark, prompting participants to decide (privately) in which of the two intervals the target had occurred. Dyad members did not consult over these initial decisions and the question mark remained on screen until both had responded. The participant using the keyboard responded by pressing “N” for the first interval and “M” for the second; the participant using the mouse used the left click for indicating the first and right click for the second interval. Individual decisions were then displayed, so that both dyad members were informed of their own and their partner’s decision. The keyboard participant’s decision was displayed in blue text, and the mouse participant’s decision was displayed in yellow text. Vertical locations of the blue and yellow text were randomised to avoid spatial biasing.

If the individual decisions disagreed, a joint decision was requested and one dyad member was selected to enter the decision. The person declaring the joint decision was determined according to the trial number: one dyad member (keyboard user) declared joint decisions on odd trials and the other (mouse user) on the even trials. As a consequence, because disagreements happened randomly, we did not have full control over exactly how many trials each participant would arbitrate. However, the assignment of arbitration by the odd/even trials ensured that, overall, both participants declared the joint decision equally frequently. The colour of the request text indicated which dyad member was to enter the decision (blue: keyboard participant, yellow: mouse participant). Dyads were free (and encouraged in the instructions) to verbally discuss their joint decision with each other for as long as they wanted.

In agreement trials, feedback was provided immediately after the individual decisions had been entered. In disagreement trials feedback was provided immediately after the joint decision had been entered. The feedback words, “Correct” or “Wrong”, were displayed in blue text for the keyboard participant, in yellow text for the mouse participant, and in white text for the joint decision. The feedback remained on screen until the next trial was initiated by the participant responding with the keyboard, after coordinating with their partner. The participants set the pace of the experiment’s progress, dependent upon the length of their discussions. Time stamps for all decisions (individual and dyadic level) were recorded relative to the start of the trial as well the beginning of the experiments.

### Data analysis

Psychometric functions were constructed for each observer [29], and for each dyad, by plotting the proportion of trials in which the oddball was reported in the second interval against the contrast difference at the oddball location (the contrast in the second interval minus the contrast in the first) (Fig 2D). The psychometric curves consisted of a cumulative Gaussian function whose parameters were bias, *b*, and variance, *σ*^2^. To estimate these parameters, a probit regression model was employed using the ‘glmfit’ function in MATLAB (release 2013b, The MathWorks Inc., Natick, Massachusetts, United States). A participant with bias *b* and variance *σ*^2^ would have a psychometric curve, denoted *P*(Δ*c*), where Δ*c* is the contrast difference between the second and first presentations, given by

$$P\left(\Delta c\right)=H\left(\frac{\Delta c+b}{\sigma }\right)$$ (1)

where *H*(*z*) is the cumulative normal function, and *z* is a general variable, to be replaced by $\frac{\Delta c+b}{\sigma }$ in our case:

$$H\left(z\right)\equiv \int_{-\infty }^{z}\frac{dt}{\sqrt{2\pi }}e^{-\frac{t^{2}}{z}}$$ (2)

The psychometric curve, *P*(Δ*c*), corresponds to the probability of choosing the second interval. Thus, a positive bias indicates an increased probability of reporting the target in the 2^nd^ interval (and thus corresponds to a negative mean for the underlying Gaussian distribution). The maximum slope of the psychometric curve, denoted *s*, is related to the variance via

$$s=\frac{1}{\sqrt{2\pi \sigma^{2}}}$$ (3)

A large slope indicates small variance and thus highly sensitive performance. Using this measure, we quantified individual participants’ as well as the dyad’s sensitivity: *s*_*Max*_ for the more sensitive participant, *s*_*Min*_ for the less sensitive participant, and *s*_*Dyad*_ for the dyad sensitivity. We defined ‘collective benefit’ as the difference between the dyad sensitivity and the best dyad member sensitivity: *S*_*Dyad*_ − *S*_*Max*_. A collective benefit value above 0 would indicate that the dyad managed to gain an advantage over its better observer. Values below 0 would indicate that collaboration was counterproductive and that the dyad did worse than its more sensitive member.

To examine effect size we used Cohen’s D [30]during 2-tailed independent sample t-tests comparisons between oxytocin and placebo groups, and Eta Squared for estimating effect size when using ANOVA and ANCOVA [31].

We examined the effect of oxytocin on proportion of disagreement trials in which the participant followed his own initial opinion. It is not trivial to statistically evaluate changes in individual proportions, or percentages, data. Such data are not normally distributed and confound to be between 0 and 1. A useful approach to evaluate proportions is analysis of covariance (ANCOVA) with the proportion denominator as covariate [32]. In our case the covariate is the number of disagreement trials arbitrated by each participant, the dependent variable is the number of disagreement trials in which the participant followed his initial opinion. The main ANOVA effects are treatment (oxytocin/placebo) and role (better/worse member). This analysis essentially fits linear models between dependent variables and covariates under each condition, and compares the slopes between them. Significant changes in slopes (i.e. significant ANCOVA effects) indicate that there is difference in proportions between groups.

### Questionnaires

General mood affect was measured throughout the experiment using a multidimensional mood questionnaire (based on [33], see Supporting Information for full questionnaire) providing a control for the effects of mood, attention, and wakefulness. The questionnaire comprised of 16 items that were rated on 7-point bipolar adjective scale (S2 Table). Each participant completed the mood questionnaire at three intervals across the experiment: at the start of the experiment, after the 20-minute rest period post-inhalation (right before the practice session), and at the end of the experiment. Comparison of the mood questionnaire results between groups did not show any significant effect (S3 Table).

## Results

**Fig 3:**
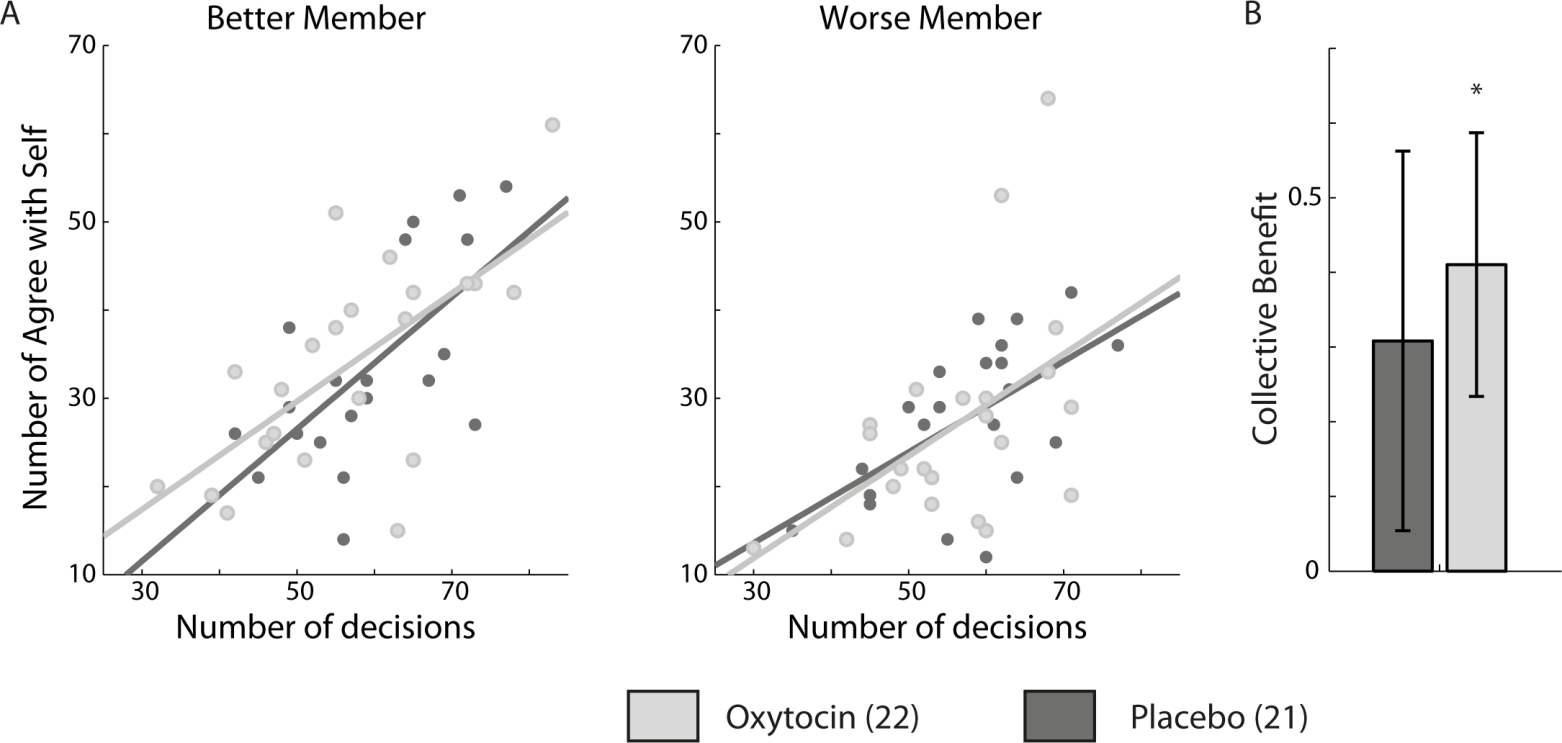
Oxytocin effect on egocentric bias collective benefit. (A) When announcing the joint decision participants could keep their original choice or go with the other member’s decision. We used ANCOVA model to estimate the relation between total number of joint decisions made by a participant and the number of egocentric (agree with self) joint decisions. This relationship is captured by the slope and intercept estimated by the ANCOVA model: steeper slope indicates an egocentric inclination, i.e. tendency to not change one’s mind during disagreements. We did not find a significant treatment effect, and no difference in slopes between worse and better members. (B) We fitted psychometric curves to the data from the entire experiment duration, estimating the dyadic sensitivity and the individual sensitivities of dyad members (*s*_*Dyad*_, *s*_*Max*_, *s*_*Min*_). Collective benefit is the difference between the dyadic sensitivity and the better dyad member (Sdyad-Smax). Under oxytocin dyads’ collective benefit was significantly higher than 0 (p = 0.026), but not under placebo (p = 0.2). However, collective benefit was not significantly different between oxytocin and placebo.

We hypothesised that oxytocin may have an effect on the participants’ willingness to change their minds during disagreements. On average, oxytocin dyads performed 310 ± 37 (mean ± std) trials and placebo dyads performed 314 ± 16 (p = 0.67) trials. Number of disagreements for oxytocin and placebo dyads were 112 ± 22, and 117 ± 17 respectively (p = 0.5). Each participant declared the dyad’s joint decisions in approximately half of the disagreement trials (keyboard participants: 57 ± 9.45; mouse participants 57.83 ± 12.2 times; paired t-test p = 0.55). When declaring the joint decisions participants could adhere to their original choice or switch to the other dyad member’s decision. We examined the relation between the number of times participant kept their original view and the overall number of joint decisions they arbitrated. We used analysis of covariance (ANCOVA) where the numbers of egocentric decisions (keeping one’s original decision) during disagreements were the dependent variable, and the total number of joint decisions declared by the participant served as covariates. The main effects were treatment (oxytocin/placebo) and role (better/worse member). ANCOVA analysis therefore fitted linear models between dependent variables and covariates under each condition, and compared the slopes between conditions. Steeper slopes indicated egocentric decisions; tendency to not change one’s mind during disagreement. The slopes were not significantly different across treatments, not for better members (F(1,39) = 0.29, p = 0.59), and not for worse members (F(1,39) = 0.05, p = 0.82), and not between better and worse participants (F(1,82) = 0.3, p = 0.58) (Fig 3A). Oxytocin did not induce overall change in allocentric/egocentric bias.

Next we examined the overall changes in oddball search performance using the psychometric curves fitted to the individuals and dyads. The fitted slopes, indicating sensitivity of visual search, were not significantly different between treatments for worse members (mean ± std, Oxytocin: 2.63 ± 1.3, Placebo: 2.98 ± 1.14, t(41) = 0.95, p = 0.34), better members (Oxytocin: 4.15 ± 1.24, Placebo: 4.69 ± 1.44, t(41) = 1.3, p = 0.2) and for dyadic decisions (Oxytocin: 4.56 ± 1.61, Placebo: 5 ± 1.09, t(41) = 1.03, p = 0.3). Infliction point, indicating bias towards one or the other choice option, were not statistically different from zero for worse members (mean ± std, 0.01 ± 0.08, t(42) = 1.3, p = 0.2), better members (-0.01 ± 0.08, t(42) = 0.97, p = 0.33) and for dyadic decisions (0 ± 0.045, t(42) = 0.33, p = 0.74).

Consistently with previous works using this paradigm [12,22], we examined collective benefit under oxytocin and placebo by fitting psychometric function to the data from all trials. We did not detect a decrease in collective benefit under oxytocin (mean ± SEM; oxytocin: 0.41 ± 0.17; placebo: 0.3 ± 0.24; t(41) = 0.34; p = 0.73) (Fig 3B). On the contrary, we found a hint for increased collective benefit under oxytocin as it was significantly different from 0 under oxytocin (t-test; t(21) = 2.37; p = 0.026, Effect size (mean/std) 0.50) but not under placebo (t(20) = 1.24; p = 0.23). This analysis did not provide evidence for decreased collective benefit under oxytocin, consistent with the absence of ego/allocentric bias, but a hint of advantage for dyadic decisions under oxytocin was observed.

In order to further examine this hinted effect we performed an exploratory time dependent analysis. When administered intranasally, the pharmacokinetics of oxytocin in the central nervous system are assumed to be similar those of vasopressin [13,34,35], and reach plateau 50 minutes after intranasal administration [36]. A number of previous studies using intranasal oxytocin found their peak effects spanning a 10-minute period some 50 minutes after administration [15,37,38]. On this basis we examined the impact of oxytocin in different time windows from administration, hypothesizing that oxytocin effect will be more apparent towards the end of the session. As mentioned in the methods section, we used a short waiting period of 25 minutes before commencing the task. We used it to allow our dyads enough time (35 minutes) to perform at least 128 trials before the peak of oxytocin effect hour after administration. As it turned up, only one dyad completed only 128 trials within 35 minutes, while the rest of the dyads were quicker, finishing 224 trials on average. We therefore were able to examine collective benefit in three 12 minutes time windows, each containing 74 ± 13 (mean ± std) trials.

**Fig 4:**
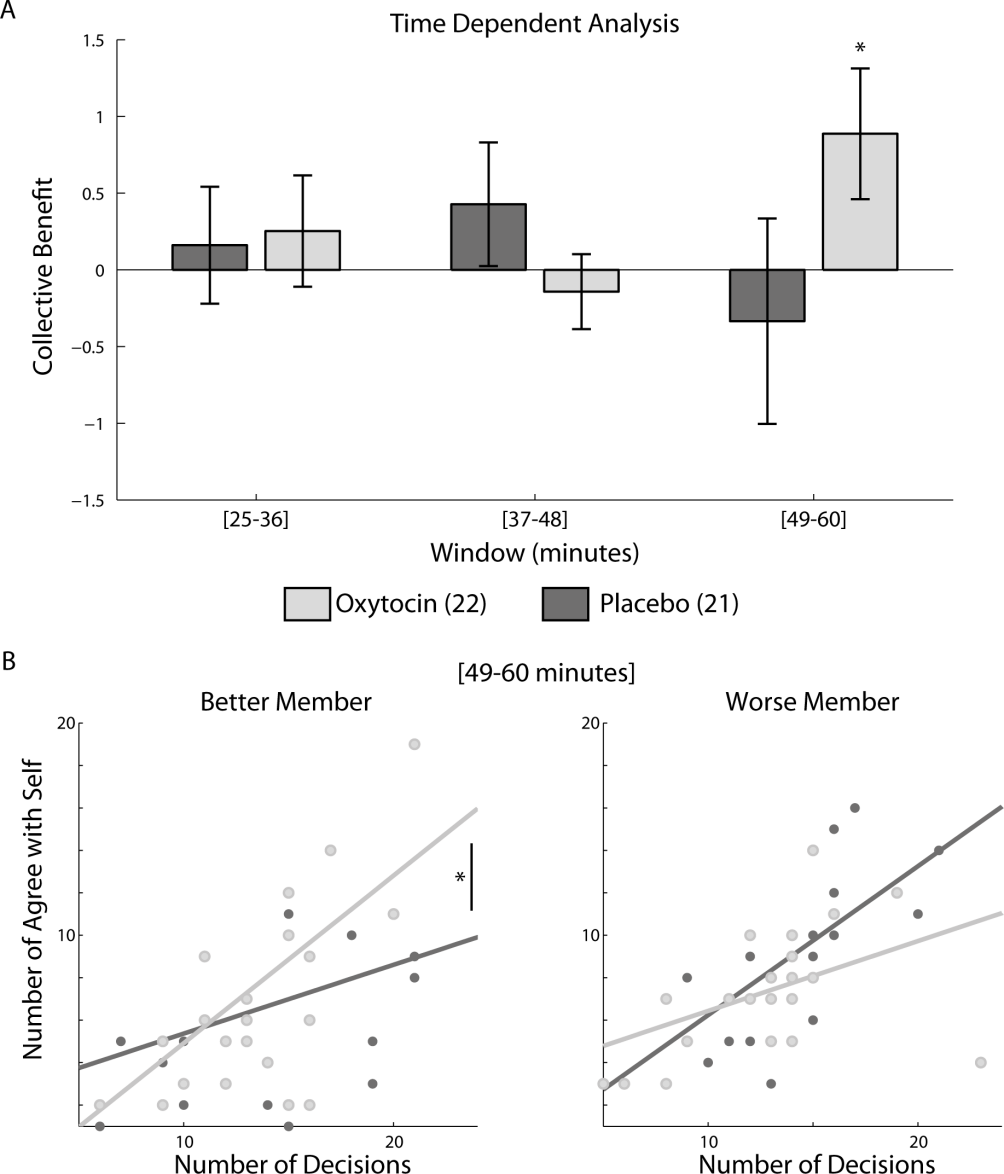
Time dependent analysis: overcoming equality bias under oxytocin. (A) We used an exploratory time dependent analysis of collective benefit, to examine whether oxytocin effect is dependent on time from administration. We estimated collective benefit in three 12 minutes time windows, with the last window in the expected time window for oxytocin effect (49–60 minutes after administration). A significant interaction effect was found (Treatment X Time F(2,82) = 3.14 p = 0.048). This interaction effect depends on the marked increase in collective benefit under oxytocin in the [49–60] minutes, the only time collective benefit was significantly higher than 0 (p = 0.045). (B) The relation between number of egocentric joint decisions and number of overall joint decisions made by the better dyad members (left panel), and the worse dyad members (right panel), during the peak in collective benefit under oxytocin. In this time window, 49–60 minutes after administration, a significant interaction is seen (p = 0.008) as better members become more egocentric (p = 0.048) and worse members become less egocentric (p = 0.1), both overcoming equality bias.

Our exploration of collective benefit over time showed that the observed advantage in collective benefit under oxytocin was temporally localized to the end of the session, during the expected peak in oxytocin effect (Fig 4A). This was captured by the interaction effect (Treatment x Time) of a mixed effects repeated measures ANOVA (F(128,2) = 3.14, p = 0.045, Effect size (Eta squared) 0.07). In addition the only time collective benefit was significantly positive was during the 49–60 minutes window under oxytocin (t(21) = 2.13, p = 0.045, Effect size (Cohen’s D) 0.47).

We extended this exploratory time dependent analysis to ego/allocentric bias during joint decisions displayed by our participants, examining ANCOVA slopes during the three time windows. Within each time window, the number of disagreement trials was 27.13 ± 6.75 for oxytocin dyads and 28.77 ± 7.5 for placebo dyads (p = 0. 38). Keyboard participants declared the joint decisions 14 ± 4.3 (mean ± std) times per window and mouse participants declared the joint decisions 13.85 ± 3.9 (mean ± std) times per window (paired t-test p = 0.5). In the first time window, 25–36 minutes after administration, egocentric slopes were not significantly different across treatment and dyad members. During the second and third time windows, 37–48 and 49–60 minutes after administration better dyad members show increased egocentric joint decisions under oxytocin compared with placebo ([37–48]: F(39,1) = 9.29, p = 0.004, Effect size (Eta squared) 0.24 [49–60]: F(39,1) = 4.16, p = 0.048, Effect size (Eta squared) 0.1). This indicates that better members overcome equality bias correctly ignoring the worse members’ opinion. It is only in the third time window, 49–60 minutes after administration, that the worse members show a trend towards decreasing egocentric decisions under oxytocin, (F(39,1) = 2.77, p = 0.1), resulting in overall significant interaction effect (F(82,1) = 7.36, p = 0.008, Effect size (Eta squared) 0.09) (Fig 4B). This interaction shows that oxytocin effected dyad members differently according to their relative performance level: better members become more egocentric and worse members less egocentric.

## Discussion

We tested the effect of oxytocin on collective decision-making and the collective benefit accrued by individuals. Previous studies of collective decision making using the same experimental paradigm as the one used here showed that dyad members suffer from equality bias [11]. Under this bias better dyad members tend to overweigh the opinions of the worse member, and vice versa, resulting in suboptimal collective benefit. Under testosterone better members overcome this bias to become more egocentric, but worse members also become more egocentric, resulting in further decrease of collective benefit [12]. We hypothesised that oxytocin will have the opposite influence, pushing both dyad members to be less egocentric and similarly resulting in reduced collective benefit. However, our analysis did not show such reduction, but hinted towards advantage in collective benefit under oxytocin. To further examine this result we used an exploratory time dependent analysis, inspired by the pharmacokinetic properties of intranasal oxytocin administration, suggesting peak activity 50 minutes after oxytocin administration. We found that advantage in collective benefit under oxytocin was delayed, and temporally localized to the 49–60 minutes window after administration. This delayed effect was accompanied with release from equality bias under oxytocin, as better members were less likely to follow the worse member’s view, and worse members more likely to follow the better member. This effect was not observed in the standard, time invariant analysis.

There are a number of important differences between our task and design and previous studies looking at social effects of oxytocin in decision-making. Rather than employing neuroeconomic paradigms that contrast competition and cooperation through monetary exchange [15,16,21], we used a visual perception task free from any element of conflict of interest [22] and focused on a reduction of decision uncertainty by social interaction. The task was optimised to detect individual and group performance level independently, and to infer collective benefit. In addition, measures such as the egocentric/allocentric bias can be calculated to investigate the processes underlying joint decision making.

Another important difference between our study and previous works is the fact that we examined pairs of face-to-face interacting participants making joint decisions, while previous studies examined one participant at a time interacting with virtual or computer-based partners [15,39–41]. Our participants had access to social cues from the other dyad member to make inferences about the partner’s reliability and performance level. Such cues are not available when playing against a virtual player. Based on our lab’s previous experience and in order to be able to compare these results with previous works [12,23], we allowed the dyad members to freely discuss their views before making joint decisions. Previous investigation of the content and style of discussions in similar experiment (but without pharmaceutical intervention) showed that more successful dyads were able to better align the terms and words they used to describe their confidence level regarding the target location [42]. Here we did not record the content of discussions, and we cannot evaluate the idea that oxytocin dyads may have had more efficient discussions and converged to more similar confidence expressions. We recorded the duration of the discussions and did not observe any difference (oxytocin: 7103 ms, placebo: 7422 ms, p = 0.71). No difference was observed in the time dependent analysis (oxytocin: 6389ms, placebo: 6886ms, p = 0.56), although both groups displayed reduction in length of discussion as the experiment proceeded.

Finally, our design demands considerably more trials than standard economic games do in order to fit psychometric curves. We overestimated the amount of time it would take participants to complete 128 trials, and used a shorter rest period (25 minutes) than usually used (50 minutes) before starting the experiment [15,37,38]. We ended up with more trials done within one hour from administration than expected, which allowed us to carry an exploratory time dependent analysis, revealing the dynamics of exogenous oxytocin’s impact on collaboration over time. This time-dependent analysis was inspired by previous estimates of the pharmacokinetic properties of intranasal oxytocin administration [13,34–36,43,44] and more recent findings of behavioural impacts of oxytocin that emerged around 50–60 minutes after administration [45]. We acknowledge that the pharmacokinetics of intranasal oxytocin administration are still the topic of debate, while direct measures of oxytocin in cerebrospinal fluid (CSF) are sparse and inconsistent [35,43,46,47]. The behavioural effects we observed using the time window analysis indeed peaked during the expected peak of oxytocin effect, 49–60 minutes after administration, in agreement with a need to delay the start of the experimental conditions [15], with the pharmacokinetics of intranasal oxytocin administration [44], and other reported behavioural effects [45]. However, it remains possible that the timing of the effect observed here was determined by the social nature of our task, or by its interaction with the pharmacokinetics of oxytocin. It is possible that oxytocin facilitated the social learning process by which participants gradually learned who is better and who is worse. Under placebo participants may have failed to learn who the better member is even though feedback was provided. The results should therefore not be taken as a clear indication of the exact temporal effect of oxytocin on behaviour. One way in which these hypotheses could be separately tested would be to run the experiment with longer waiting period, starting the experiment proper 50 minutes after administration for shorter duration (one time window of 15 minutes). If oxytocin enhances a slow but stable social learning process it may have no effect on collective benefit when social interaction is short. If oxytocin effect is temporally locked and transient, then it may enhance collective benefit even with no previous history of interaction.

Our results highlight the role dependent effect for oxytocin such that its effects were contingent upon a subject’s relative performance level. Under oxytocin dyad members settled disagreements according to performance level within the dyad, resulting in a disposition for the better dyad to adhere to their initial decisions and the less sensitive member to change their mind. We suggest that enhanced monitoring of own and other’s performance may underlay the effect of oxytocin on collective decision making. When performing a shared or collaborative task one needs to monitor his own performance, his partner’s performance and the task at hand. Here, in order to learn who is better and who is worse one has to monitor his and other’s individual responses, feedback and stimuli. Oxytocin may enhance such monitoring processes and social learning, giving rise to the observed role dependent adjustment of behaviour. We speculate that this may be mediated by the medial prefrontal cortex (mPFC), area associated with monitoring of errors for oneself and others [48–55], and an area greatly affected by oxytocin [56–62].

Enhanced monitoring of self and other’s performance and errors may contribute to accurate estimation of one’s relative performance level within the dyad. Dyad members adjusted the number of mind changes during disagreements according to their relative performance, and were less affected by equality bias [11]. This role dependent effect is in line with the more general notion of context dependent oxytocin effect [63]. Bartz et al. [63] suggested that oxytocin may not exert a single stable effect, as implied for example by the term “love hormone”, which implies the same effect on behaviour regardless of task, person, and social context. Instead, our data suggest its effect is highly context and person dependant, and this might result in apparently contradictory behaviours under different circumstances. For example, while oxytocin has been shown to increase trusting behaviour [15], this effect disappears if the potential trustee is portrayed as untrustworthy [64] or is unknown [65].

To conclude, using an exploratory time dependent analysis we observed a delayed and role-dependent effect of oxytocin on collective decision making. Under oxytocin participants were released from equality bias and accrued higher collective benefit 50 minutes after administration. We suggest that this effect is mediated by enhanced monitoring of own and other’s performance. Enhanced monitoring allowed participants to recognize their relative role in the dyad increasing the efficiency of collective decisions during disagreements. The role dependent effect of oxytocin observed here emphasises the importance of context in effects of exogenous oxytocin, as it may produce contradictory behaviour under different circumstances.

## Supporting Information

S1 Dataset Two CSV files contain the data from 43 dyads in our two analysis approaches: whole data and time analysis. Whole date table columns are: dyadic treatment (Oxytocin = 1, Placebo = 0), dyad slope (see Methods), better member (Max) slope, worse member (Min) slope, total number of trials performed, number of disagreement trials, number of disagreement arbitrated by Max, number of mind changes during disagreements arbitrated by Max, number of disagreement arbitrated by Min, number of mind changes during disagreements arbitrated by Min, time from beginning of experiment in minutes (experiment starts 25 minutes after administration). Time analysis table include data from the three 12 minutes time windows: win1 [25–36 minutes from administration], win2 [37 48], win 3 [49 60]. Its columns are: treatment, dyad slope (three windows), Max slope (three windows), Min slope (three windows), Number of trials per window, Number of disagreements (three windows), Number of disagreement arbitrated by Max (three windows), Number of Mind Changes by Max (three windows), Number of disagreement arbitrated by Min (three windows), Number of Mind Changes by Min (three windows).
(ZIP)

S1 Table CONSORT checklist. This is a checklist of the 25 items that must be reported in a Randomized Clinical Trial Report according to the CONSORT (CONsolidated Standards of Reporting Trials) 2010 standard [66]. See also: http://www.consort-statement.org/consort-2010.
(DOCX)

S2 Table Mood Questionnaires. A copy of the mood questionnaire filled by the participants. Questionnaires were completed at the start of the experiment, after the 20 minute rest period (prior to commencing the task), and at the end of the experiment. Each item was answered by marking a point on a 1–7 Likert scale.
(DOCX)

S3 Table Changes in mood across treatments. Difference scores in ratings between questionnaires 2 and 1, and between questionnaires 3 and 1 are shown for each of the subjective state items. Rating differences were compared across the treatment conditions using independent t-tests.
(DOCX)
