# Supplementary material for: Oxytocin Effect on Collective Decision Making: A Randomized Placebo Controlled Study
Source: PLoS One. 2016 Apr 12;11(4):e0153352. doi: 10.1371/journal.pone.0153352 (PMC4829266; doi:10.1371/journal.pone.0153352)
Supplement: S2 Table — A copy of the mood questionnaire filled by the participants. Questionnaires were completed at the start of the experiment, after the 20 minute rest period (prior to commencing the task), and at the end of the experiment. Each item was answered by marking a point on a 1–7 Likert scale. (DOCX) [file pone.0153352.s003.docx]

S2 Table: Mood Questionnaire

A copy of the mood questionnaire filled by the participants. Questionnaires were completed at the start of the experiment, after the 20 minute rest period (prior to commencing the task), and at the end of the experiment. Each item was answered by marking a point on a 1 - 7 Likert scale.

| For each scale below check the box which represents how you feel at this moment. The ends of each scale are to represent the “most” that you have ever felt in your life. | | | | | | | | |
| --- | --- | --- | --- | --- | --- | --- | --- | --- |
|  | 1 | 2 | 3 | 4 | 5 | 6 | 7 |  |
| Alert |  |  |  |  |  |  |  | Drowsy |
| Calm |  |  |  |  |  |  |  | Excited |
| Strong |  |  |  |  |  |  |  | Feeble |
| Muzzy |  |  |  |  |  |  |  | Clear Headed |
| Well-Coordinated |  |  |  |  |  |  |  | Clumsy |
| Lethargic |  |  |  |  |  |  |  | Energetic |
| Contented |  |  |  |  |  |  |  | Discontented |
| Troubled |  |  |  |  |  |  |  | Tranquil |
| Mentally Slow |  |  |  |  |  |  |  | Quick Witted |
| Tense |  |  |  |  |  |  |  | Relaxed |
| Attentive |  |  |  |  |  |  |  | Dreamy |
| Incompetent |  |  |  |  |  |  |  | Proficient |
| Happy |  |  |  |  |  |  |  | Sad |
| Antagonistic |  |  |  |  |  |  |  | Friendly |
| Interested |  |  |  |  |  |  |  | Bored |
| Withdrawn |  |  |  |  |  |  |  | Sociable |
