# Supplementary material for: Oxytocin Effect on Collective Decision Making: A Randomized Placebo Controlled Study
Source: PLoS One. 2016 Apr 12;11(4):e0153352. doi: 10.1371/journal.pone.0153352 (PMC4829266; doi:10.1371/journal.pone.0153352)
Supplement: S3 Table — Difference scores in ratings between questionnaires 2 and 1, and between questionnaires 3 and 1 are shown for each of the subjective state items. Rating differences were compared across the treatment conditions using independent t-tests. (DOCX) [file pone.0153352.s004.docx]

S3 Table : Changes in mood across treatments

Difference scores in ratings between questionnaires 2 and 1, and between questionnaires 3 and 1 are shown for each of the subjective state items. Rating differences were compared across the treatment conditions using independent t-tests.

|  | **Questionnaire 2 – Questionnaire 1** | | | **Questionnaire 3 – Questionnaire 1** | | |
| --- | --- | --- | --- | --- | --- | --- |
| **Bipolar adjective item** | **Oxytocin** | **Placebo** | **p-value** | **Oxytocin** | **Placebo** | **p-value** |
| Alert 1 – Drowsy 7 | 0.28 | 0.45 | 0.59 | 0.50 | 0.32 | 0.66 |
| Calm 1 - Excited 7 | -0.25 | -0.45 | 0.52 | 0.31 | -0.02 | 0.36 |
| Strong 1 - Feeble 7 | -0.11 | 0.16 | 0.17 | -0.11 | 0.16 | 0.35 |
| Muzzy 1 - Clear Headed 7 | -0.22 | -0.45 | 0.41 | -0.11 | -0.39 | 0.43 |
| Well-Coordinated 1 - Clumsy 7 | 0.28 | 0.23 | 0.85 | 0.11 | 0.25 | 0.69 |
| Lethargic 1 - Energetic 7 | -0.08 | -0.34 | 0.33 | -0.33 | -0.14 | 0.59 |
| Contented 1 - Discontented 7 | -0.11 | 0.05 | 0.49 | 0.00 | 0.20 | 0.44 |
| Troubled 1 - Tranquil 7 | 0.22 | 0.09 | 0.54 | 0.31 | 0.18 | 0.61 |
| Mentally Slow 1 - Quick Witted 7 | -0.17 | -0.16 | 0.98 | -0.14 | -0.36 | 0.51 |
| Tense 1 - Relaxed 7 | 0.00 | 0.27 | 0.37 | -0.11 | 0.18 | 0.41 |
| Attentive 1 - Dreamy 7 | 0.42 | 0.61 | 0.52 | 0.33 | 0.18 | 0.70 |
| Incompetent 1 - Proficient 7 | -0.08 | -0.14 | 0.84 | -0.44 | -0.20 | 0.32 |
| Happy 1 - Sad 7 | 0.14 | 0.18 | 0.84 | -0.03 | -0.02 | 0.99 |
| Antagonistic 1 - Friendly 7 | -0.39 | 0.02 | 0.13 | 0.06 | 0.07 | 0.96 |
| Interested 1 - Bored 7 | 0.58 | 0.57 | 0.96 | 0.69 | 0.36 | 0.29 |
| Withdrawn 1 - Sociable 7 | -0.19 | 0.00 | 0.28 | 0.28 | 0.18 | 0.71 |
